# Evaluation of pain knowledge and attitudes and beliefs from a pre-licensure physical therapy curriculum and a stand-alone pain elective

**DOI:** 10.1186/s12909-019-1820-7

**Published:** 2019-10-16

**Authors:** Sonja K. Bareiss, Lucas Nare, Katie McBee

**Affiliations:** 1 Department of Physical Therapy, School of Movement and Rehabilitation Sciences at Bellarmine University, Nolen C. Allen Building, 2001 Newburg Rd. Room 471, Louisville, KY 40205 USA; 2 Department of Neurological Surgery, University of Louisville, Louisville, USA; 3 Select Medical, Mechanicsburg, PA USA

**Keywords:** Education, Pain, Pain knowledge, Physical therapy curriculum

## Abstract

**Background:**

Adequate pain education of health professionals is fundamental in the management of pain. Although an interprofessional consensus of core competencies for health professional pre-licensure education in pain have been established, the degree of their incorporation into physical therapy curriculum varies greatly. The purpose of this study was to 1. Assess students’ pain knowledge and their attitudes and beliefs in a pre-licensure physical therapy curriculum using a cross sectional comparison, and 2. Using a sub-sample of this population, we evaluated if an elective course on pain based on International Association for the Study of Pain (IASP) guidelines had an effect on students’ knowledge and beliefs.

**Methods:**

The Neurophysiology of Pain Questionnaire (NPQ) and the Pain Attitudes and Beliefs Scale for Physiotherapists (PABS-PT) was completed by first semester (*n* = 72) and final (*n* = 56) semester doctor of physical therapy (DPT) students. Final semester students completed surveys before and after participation in an elective course of their choosing (pain elective (PE) or other electives (OE)).

**Results:**

Participation rate was > 90% (*n* = 128/140). We found mean differences in NPQ scores between final semester (3rd year) students (76.9%) compared to first semester students (64%), *p* < 0.001. Third year students showed a mean difference on PABS-PT subscales, showing decreased biomedical (*p* < 0.001) and increased biopsychosocial (*p* = 0.005) scores compared to first semester students. Only final semester students that participated in the PE improved their NPQ scores (from 79 to 86%, *p* < 0.001) and demonstrated a significant change in the expected direction on PABS-PT subscales with increased biopsychosocial (*p* = 0.003) and decreased biological scores (*p* < 0.001).

**Conclusions:**

We suggest that although core pre-licensure DPT education improves students’ pain knowledge and changes their attitudes towards pain, taking a IASP based pain elective continues to improve their pain neurobiology knowledge and also further changes their attitudes and beliefs towards pain. Therefore, a stand-alone course on pain in addition to pain concepts threaded throughout the curriculum may help ensure that entry-level DPT students are better prepared to effectively work with patients with pain.

## Background

Chronic pain affects more than 100 million Americans and is widely recognized as a major social, economic, and medical problem [1]. The steady increase in the prevalence of chronic pain is due, in part, to inadequate treatment and mismanagement of pain, which could delay healing and promote long-term undesirable changes in nervous system plasticity [2]. According to the Centers for Disease Control and Prevention, non-pharmacological therapy and non-opioid pharmacologic therapy are preferred for chronic pain [3], with physical therapy management specifically identified as a non-pharmacological option to treat pain [3]. Physical therapists already play an important role in the management of pain across multiple conditions, and the role for physical therapy services is continuing to grow as an entry-point for patients seeking treatment for pain [4]. As a result, physical therapists are a part of the first-line non- pharmaceutical management for people with pain [3]. Given that pain is often the most common reason patients seek care (by direct access or referral by another practitioner), a contemporary and thorough understanding of pain is paramount in all physical therapy education programs.

In 2011, the Institute of Medicine published a report on pain describing the need to transform health care pain education, prevention, and care. This report identified significant deficiencies in pain education across health care disciplines, including physical therapy [1]. Recently, a faculty survey report showed that in accredited physical therapy programs in the United States, the average time spent on pain education was 31 h (1.6% of total didactic education) [5, 6]. Similarly, physiotherapy students in the United Kingdom averaged 38 h of pain education (1.9% of total didactic education) [7]. Although time spent on pain education has risen significantly from 2001 [8], contact hours vary greatly among programs (5–115 h) and only 61% of respondents believed pain was adequately covered in their curriculum [6].

To address the deficits in pain education, the International Association for the Study of Pain (IASP) developed an outline of pain curricula for undergraduate and graduate health professional education, including physical therapists [9]. Curricular recommendations included education on 4 major components: I. Multidisciplinary nature of pain, II. Pain assessment and measurement, III. Management of Pain, and IV. Clinical conditions [9]. Preliminary reports for implementation of IASP curricula in dentistry, medicine, nursing, pharmacy, physical therapy, and occupational therapy are promising demonstrating improved knowledge and competency towards the treatment of pain [10–12]. Yet, despite the supportive evidence and recommendations, incorporation of these guidelines into core curriculum has been lagging [7]. Although ways to integrate pain management core competencies have been outlined for DPT, Bement et al. suggested that a stand–alone pain course could supplement the integration of pain education throughout curriculum emphasizing underlying pain science within biological and psychosocial effects of pain [13]. In a faculty survey of pain curricula in the United States, only 11 (6.6%) of the 167 physical therapy programs reported offering an independent pain course [6]. Pain science in most physical therapy programs is not explicitly addressed, and is primarily taught in foundational neuroscience and orthopedic curriculum with a biomechanical approach, organized by region or around anatomical areas – such as knee, hip, shoulder, and spine [13]. Thus, this may leave important gaps in foundational pain knowledge, assessment, and management for pre-licensure students. Given the integral role that physical therapists play in pain management as part of the interdisciplinary team, adequate training in pain mechanisms and treatment is an important part of effectively assessing and managing pain.

The objective of this study was to 1. Assess students’ pain knowledge and their attitudes and beliefs following core pre-licensure physical therapy curriculum using a cross sectional comparison, and 2. Using a sub-sample of this population, evaluate if a comprehensive pain elective course based on the IASP curricular guidelines increased students’ knowledge and changed their attitudes and beliefs regarding the management of pain.

## Methods

### Educational context

The cross-sectional study assessing students’ knowledge and attitudes and beliefs was conducted at Bellarmine University in Louisville, KY, in the United States. The institution offers a post graduate Doctor of Physical Therapy degree. Approximately 72 new students per cohort enroll every academic year. The Bellarmine DPT program is a 3 year, 9 semester program consisting of 142–146 credit hours. Students were surveyed in January (3rd year students) and May (1st year students) in the 2017 academic year.

### Participants

The study was approved by Institutional Review Board at Bellarmine University (#555). Participation in the survey was optional, and all data from participants was de-identified for reporting. This cross-sectional cohort study was conducted on 72 first and 56 final semester students of the Doctor of Physical Therapy (DPT) program at Bellarmine University. Surveys were administered face-to-face prior to scheduled class time. Prior to survey administration students were given verbal information that their participation in the survey would not impact their scores or performance in any course or standing in the program. Students were also informed that their participation in the survey was completely voluntary. After these instructions were given, consent to participate was implied by participation in the survey. Four individuals were excluded from the survey because they were involved in collecting and scoring the data from the questionnaires. First year (first semester) DPT students completed surveys on their first day of class, prior to their beginning introductory coursework. Third year (final semester) DPT students completed the surveys before and after participation in a two credit hour (30 contact hour) elective course of their choosing. At the time the survey was administered, final semester students had completed 135 credit hours, and the elective was the last class of their didactic coursework before entering their final 12 weeks of clinical rotation (for a total of 42 weeks) prior to graduation.

#### Variables

The main variable was the result of the Neurophysiology of Pain Questionnaire (NPQ) and Pain Attitudes and Beliefs Scale for Physiotherapists (PABS-PT). Secondary variable was participation in a pain elective.

#### Questionnaires

The Neurophysiology of Pain Questionnaire (NPQ) was used to assess students’ knowledge about pain neurobiology [14]. The NPQ was devised to assess knowledge related to the biological mechanisms that underpin the experience of pain. The NPQ contains 19 closed-ended (True or False) questions related to the neurophysiology of pain. Scoring for the NPQ is reported as the number of correct responses where correct responses value 1 point and incorrect or unanswered value 0 points. A higher score on the NPQ indicated greater understanding of pain neurophysiology. A final Yes/No question was added to the questionnaire that asked whether students had participated in additional pain education beyond their core physical therapy curriculum. The questionnaire has been evaluated for its psychometric properties and demonstrated that it had acceptable construct validity, internal consistency (Person Separation index = 0.84), and test-retest reliability [15].

The Pain Attitudes and Beliefs Scale for Physiotherapists (PABS-PT [16, 17]) was used to evaluate students’ attitudes and treatment orientation towards management of patients with low back pain (LBP). This instrument was originally developed to screen for attitudes and beliefs and treatment recommendations of physical therapists [16]. Although the PABS-PT instrument continues to be refined, psychometric properties of validity and reliability have been found to be satisfactory [18]. Scores for the PABS-PT were calculated as previously described by the questionnaire developers which included brief simple summation of the items from each subscale [17]. The PABS-PT originally contained 31 item questionnaires [17], and has since been revised to 19 items [16]. Participants are asked to rate items (statements) about LBP on a 6 point Likert scale from ‘Totally disagree’ = 1 to ‘Totally agree’ = 6. Items are categorized into two subscales, either ‘biomedical’ or ‘behavioral’, and then each subscale is summed to produce a score. The biomedical scales consists of 10 items (score range: 10–60) and behavioral scales consist of 9 items (score range: 9–54). The biomedical subscale is described as orientation in which physical therapists believe in a biomechanical model of pain, where there is a direct relationship between pain and specific tissue pathology. Any missing data on this questionnaire was handled as previously reported [19]. If one question/value was missing from a subscale, a mean score based on the remaining values was submitted. The behavioral orientation is where physical therapists believe in a biopsychosocial model of pain in which pain does not have to be a consequence of tissue damage, but is influenced by psychological, social, and behavioral factors [16]. Higher scores on each factor indicate a stronger biomedical or behavioral orientation, respectively.

#### Elective course on pain

Final semester DPT students were selected from one of the following elective courses: Pain Mechanisms and Treatments (pain elective, PE, *n* = 30), Aquatics or Neurology (other electives, OE, *n* = 26). All courses were delivered within the same period of time (2.5 weeks) and consisted of 2 credits (30 contact hours). Students participating in the OE served as a control group. The PE was designed as a comprehensive final semester pain course following the IASP pain curriculum guidelines addressing the multidimensional nature of pain, pain assessments and screening tools, multimodal management of pain, and clinical conditions [9]. Course content within these four areas was modeled from an example stand-alone pain course proposed by Bement et al. and the international consensus of core competencies for prelicensure education in pain management [9, 13], with didactic lecture material based on the revised edition of Sluka’s *Mechanisms and Management of Pain for the Physical Therapist* [20]. The course also consisted of guided laboratory activities, case presentations, and discussion with an interprofessional pain panel (comprised of a physical therapist, physician, nurse, and psychologist, all of whom specialize in the management of chronic pain). Independent from the questionnaires, assessment strategies for the PE course included a complex case study presentation and a written exam. To account for potential bias, there was no particular reference to any questions on the NPQ or PABS-PT during the pain elective.

#### Data analysis

Data from the questionnaires were analyzed using Graph Pad Prism 7. Descriptive statistics were reported as mean (standard deviation) and the Shapiro-Wilk test was used to determine if the data was normally distributed. NPQ results were expressed as means (percentage of correct responses). Comparisons were accomplished using independent t-tests (1st vs 3rd year) and dependent t-tests (3rd year pre and post elective session scores) for both questionnaires. Data were expressed as means (standard deviation) unless otherwise indicated with significance set at *p* < 0.05. For both questionnaires, data entry was completed by an investigator blinded to study design.

## Results

### Cross sectional comparison of 1st and 3rd year students

Out of the total students enrolled in the first semester (1st year) and final semester (3rd year) of the program (*n* = 140), greater than 90% participated in the survey (*n* = 128). Of these, 72 (100%) 1st semester students and 56 final (82%) semester students participated. Reasons for lower participation rate from final semester students were related to absence due to illness and/or participation in an international study abroad during survey administration. The subjects’ demographic characteristics and mean (standard deviation, SD) questionnaire scores of the 128 students that completed the survey are presented in Table 1. First semester DPT students mean age (SD) was 21.19 (2.5) years with 72.0% female, final semester students mean age was 25.5 (1.7) years with 71.4% female..

**Table 1:** Student demographical data, NPQ, and PABS-PT scores between 1st year and 3rd year students

	1st YEAR	3rd YEAR, Prior to Elective
N	72	56
Sex:		
M # (%)	M 27 (38.0%)	M 16 (28.6%)
F # (%)	F 45 (62.0%)	F 40 (71.4%)
Age (years)	22.9 (2.5)	25.5 (1.7)
NPQ Score	64.0% (10.1)	76.9% (8.9), *p* < 0.001
PABS-PT (subscales)		
Biomedical	37.10 (4.5)	33.78 (5.8), *p* < 0.001
Biopsychosocial	32.99 (3.5)	34.85 (3.7), *p* = 0.005

Data are presented as mean (standard deviation) except for sex. Neurophysiology of Pain Questionnaire (NPQ) scores indicate percentage of correct responses, Pain Attitudes and Beliefs Scale for Physiotherapists (PABS-PT) Biomedical and Biopsychosocial mean subscale scores. Independent t-test was used to compare between the 1st and 3rd year students

The students’ knowledge of pain showed that 3rd year students’ mean scores were significantly higher on the NPQ76.9% (95% confidence interval [CI] 74–6-79.3) compared to first year/semester students 64.0%, (CI 61.7–66.4; *p* < 0.001), with an average difference of 13% between cohorts. Attitudes and beliefs assessed with the PABS-PT questionnaire demonstrated statistically significant mean group differences between 1st and 3rd year students. Biomedical subscale showed means decreasing from 1st year 37.1 (CI 36.1–38.3) to 3rd year 33.8, (CI 32.2–35.4), *p* < 0.001; whereas mean differences increased on the biopsychosocial subscale from 1st year 32.9 (CI 32.2–33.8) to 3rd year 34.9 (CI 33.8–35.9), *p* = 0.005 (Table 1).

### Comparison of final semester students pre and post-elective

Third year (final semester) students enrolled in either the pain elective (PE, *n* = 30) or other elective (OE, *n* = 26) of their choosing. Baseline scores of final semester students prior to electives, showed NPQ scores were slightly higher in the PE 78.7% (7.1) than the OE 73.5% (9.2), *p* = 0.05. However, mean scores on the PABS-PT biomedical (PE 33.2 (6.2) and OE 34.4 (5.4), *p* = 0.20) and biopsychosocial scores (PE 35.3 (3.1) and OE, 34.4 (4.3), *p* = 0.80) revealed baseline scores between the groups were not different (Table 2). To evaluate the effect of the PE on students pain knowledge and attitudes towards pain, the NPQ and PABS-PT were administered prior to and immediately following completion of the 3rd year electives within the same 2.5 week period. Pre- and post-NPQ scores for the elective courses showed that only students taking the PE improved their scores from 78.7% (7.6) CI 75.9–81.6 to 86.0% (7.4), CI 82.4–88.1, *p* < 0.001, whereas students taking OE showed no difference in their pre 73.5%, (9.5), CI 72.9–81.2 and post-elective 76.1% (9.4) CI 75.3–83.5, *p* = 0.40) NPQ scores. PABS-PT scores had statistically significant changes between pre and post-PE elective scores, showing increases in biopsychosocial scores (*p* < 0.001), and decreases in biomedical (*p* = 0.003) (Table 3). There were no statistically significant differences in either PABS-PT subscale score between pre and post-OE training (biomedical *p* = 0.49, biopsychosocial *p* = 0.95).

**Table 2:** NPQ scores pre and post electives

Pain Elective (PE), *N* = 30				Other Elective (OE), *N* = 26			
	Mean (SD)	Min	Max		Mean (SD)	Min	Max
Pre	78.7 (7.1)	57.9	100	Pre	73.5 (9.5)	57.9	94.7
Post	86.0 (7.4)**	68.4	94.7	Post	76.1 (9.4) ns	63.2	94.7

Values indicate mean percentage of correct responses (SD). Minimum (min) and maximum (max) scores for Pain Elective (PE) and Other Elective (OE). Paired t-tests for pre and post- NPQ scores for PE and OE, **p < 001

**Table 3:** Mean subscale scores on the PABS-PT pre- and post-elective Pain Elective (PE) or Other Elective (OE)

PABS-PT		Pain Elective (PE), *N* = 30			Other Elective (OE), *N* = 26			
Subscale	Time	Mean (SD)	95% confidence interval		Time	Mean (SD)	95% confidence interval	
			Lower	Upper			Lower	Upper
Biomedical	Pre	33.2 (6.2)	30.8	35.7	Pre	34.4 (5.4)	32.1	36.6
	Post	26.1 (5.6) *p < 0.001	23.8	28.4	Post	33.8 (4.0) ns, *p* = 0.49	32.1	35.4
Biopsychosocial	Pre	35.3 (3.1)	34.1	36.5	Pre	34.4 (4.3)	32.7	32.9
	Post	38.3 (3.5) *p = 0.003	82.4	39.7	Post	34.5 (3.6) ns, *P* = 0.95	32.9	35.9

Pain elective pre-post paired t-test for each subscale, other elective pre-post paired t-test for each subscale

### Effect of previous pain education

**Fig. 1:**
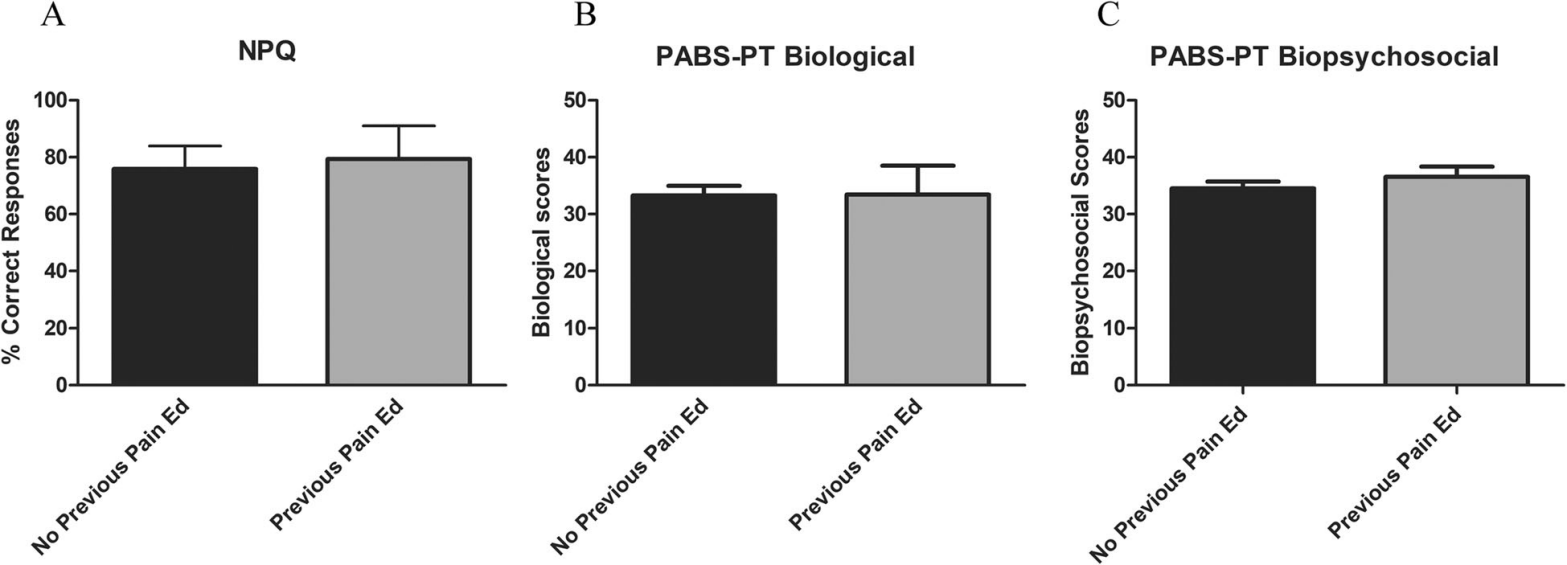
Comparison of previous pain education on NPQ and PABS-PT scores. Legend: Comparing 3rd year students with (*n* = 11) and without previous pain education (*n* = 45) (**a**). NPQ (**b**). PABS-PT biomedical subscale (**c**). PABS-PT biopsychosocial subscale. Scores presented as mean (standard deviation) with 95% confidence intervals. Independent t-test for NPQ and PABS-PT. There was no difference between those that had taken a previous pain elective and those that did not

Given the recent reports on different educational types and formats [12, 14, 21] that students might participate in as part of their clinical experience or conference attendances, a final survey question added to the NPQ asked if students had previously attended any pain education course(s). Students simply answered “yes” or “no” indicating if they had attended a previous pain education course through continuing education courses, workshops, or conference sessions. There were no reports of prior pain education from the first year students. However, 20% (11/56) of the 3rd year students reported attending previous pain education. Thus, we wanted to determine if there were differences between students that participated in previous pain education and those that did not. Post-hoc analysis on students that participated in previous pain education (*n* = 11) demonstrated slightly higher NPQ scores 79.4% (11.1), however their scores were not significantly higher compared to students without previous pain education *n* = 45, 75.9% (8.1), *p* = 0.23 (Fig. 1 a). Similarly, PABS-PT scores from students with previous pain education (*n* = 11) showed no difference biomedical orientation (previous pain education, 33.3 (5.6), no previous pain education (*n* = 45) 33.5 (7.5), *p* = 0.79; or biopsychosocial scale (previous pain education 34.5 (3.9), no previous pain education 36.5 (2.7), *p* = 0.19 (Fig. 1b, c).

## Discussion

We found that pre-licensure core curriculum improved students’ pain neurobiology knowledge and that a stand-alone elective course on pain based on the IASP curricular guidelines significantly improved students’ knowledge and changed their attitudes and beliefs on treating patients with pain.

Our findings of low (64%) baseline NPQ scores in entry level/first semester physical therapy students align well with Moseley’s original report surveying untrained therapists with NPQ scores of 61% and Hush’s recent report of a single pre-licensure physical therapy program with reported NPQ scores of 58% [14, 22]. Other groups have reported significantly lower starting scores of 41% [23], 43% [24], and 45% [21]). Although the reason for this discrepancy is unknown, it might be attributed to variations in program admissions processes or sampling of undergraduate vs. graduate entry-level programs [21, 23, 24]. Interestingly, the NPQ has recently been revised following a Rasch analysis demonstrating that several questions may perform poorly for persons with differing abilities and other questions exhibited local dependence [15]. Although we administered the original 19 point NPQ, we reanalyzed our data with the 7 questionable items excluded by the Rash analysis and determined there was no impact on our results (see Additional file 1). Since the NPQ has no cut-off value of what is a sound knowledge score, we relied on percentage scores which were comparable with other reports [14, 22].

Here we report that final semester students (prior to electives) scored 77% which is again consistent with Moseley (78%) and Hush (77%) from reports surveying trained clinicians and final semester entry-level students [14, 22], suggesting that core pre-licensure curriculum improves students’ pain knowledge, and that this knowledge was retained in their final semester. Importantly, 3rd year (final semester) students that participated in the pain elective course scored 10% higher on the NPQ (86%) than their counterparts that chose the other electives (76%), which served as the control group in this study. This small (1.94) improvement in score has also been reported in a recent study investigating the education of patients showing a 1.96 point improvement in NPQ, which was considered clinically significant in patients exposed to pain education, showing that higher levels of education improved NPQ score compared to those with lower education levels [25]. In our study, the increase in correct responses may be related to the breadth of what is taught in an IASP curriculum. Given that the NPQ seeks to measure an individual’s view of whether pain is caused by and directly linked to tissue health, this indicates that the IASP curriculum improves this understanding. Therefore, we demonstrate that student knowledge scores on the NPQ could be enhanced after completion of a stand-alone comprehensive pain course modeled after IASP curricular guidelines for physical therapy.

In this study we also found that an IASP based pain course changed students’ attitudes, by increasing biopsychosocial scores and decreasing biomedical treatment orientation scores. These directional shifts in attitudes and beliefs about pain were expected and are consistent with IASP based curricular reported outcomes from other health professional programs [10, 12]. Here we report a negative 7.1 point biomedical and positive 3.0 point biopsychosocial change in the PABS-PT subscales following the pain elective. These improvements were comparable with a recent report of psychologically informed training for physiotherapists, which showed negative 4.9 (biomedical), and positive 4.7 (biopsychosocial) point changes for each subscale [26]. These finding are consistent with a number of other studies that show that education can alter attitudes and beliefs about pain [21, 27], and importantly, show that that health care provider attitudes are associated with making treatment recommendations that follow clinical guidelines [16, 21, 28, 29]. Although clinical competencies were not assessed in this study, embedded IASP based pain curriculum in an Australian pre-licensure physical therapy program demonstrated a high level of clinical competencies in pain management. As such, it is tempting to suggest that knowledge and beliefs would change clinical practice. Undoubtedly, more studies are needed to examine IASP pain education on the association between knowledge, beliefs, and clinical competencies.

### Comprehensive vs. short course

The pain elective course in this study was designed to be comprehensive and modeled after the sample outline of a stand-alone course published by Bement et al. [13]. The course covered basic pain principles and science, pain assessment and management, and condition specific considerations. We found that this approach enhanced students’ pain knowledge and changed their attitudes about pain. Similarly, Strong et al. also found that a 28 h pain education course based on IASP guidelines improved students’ knowledge of pain from across a variety of backgrounds including occupational therapy, physical therapy, and dentistry [12]. This is in contrast to a number of single, short abbreviated (70–180 min) pain education formats that have been described and implemented in physical therapy curricula and continuing education workshops [21, 23, 30]. Interestingly, while most abbreviated courses improve knowledge [14, 21, 23] not all show an impact on changing attitudes and beliefs. A recent report of a United States physical therapy program found attitudes and beliefs after a 3 h pain education session were unchanged [23]. This would support that more education is likely needed to change physical therapy student’s view from biomedical/ biomechanical to biopsychosocial. Furthermore, because of the time constraints of these abbreviated courses, the full educational standards outlined by the IASP are likely to only be partially addressed [9]. This is supported by a recent study showing that time spent on pain education demonstrates a pattern in which the total number of hours of pain education within entry-level programs seems to correspond to a higher proportion of IASP pain content that is fully integrated [31]. Undoubtedly, uses of these two questionnaires are insufficient to assess all of these curricular elements covered in our pain elective. Additional assessments of clinical skills and competencies related to the four domains as outlined by the IASP, are needed to determine the extent to which pain courses address the complex and multidisciplinary nature of pain on student learning outcomes [31].

### Integration vs. stand-alone course

Students in this study were offered an elective course on pain, and thus only half the students (53%) of the cohort were exposed to standardized pain education curriculum proposed for DPT programs [9, 13]. We show that knowledge of pain neurophysiology can be changed, as can attitudes and beliefs about pain, even when DPT students are in their final semester of study following participation on a comprehensive pain elective. This also suggests that waiting until final semester may not be optimal for some students who have an interest in learning more or changing beliefs about pain. An alternate option to a stand-alone course is the explicit integration of pain competencies woven throughout a health sciences program [9]. Indeed a recent report demonstrated that embedding the IASP pain curriculum into a de novo 3-year pre-licensure DPT program improved student pain knowledge (NPQ scores) and achieved higher level of clinical competencies in pain management [22]. Interestingly those student mean scores were identical with our 3rd year (pre-elective) DPT student NPQ scores (both at 77%) [22]. Although the specific mapping of pain units and learning outcomes has not been charted for our program, students’ pain knowledge on the NPQ seems consistent between these two programs [22]. To address any unmet educational gaps in pain, our program offered a comprehensive single course solely dedicated to reviewing and expanding pain content to 3rd year (near entry-level) DPT students [10]. This also provided students with interest in developing enhanced training in pain, the opportunity to build their skills in this area of practice. Indeed our baseline NPQ scores showed slightly higher mean values in the pain elective (79%) compared to other elective (74%), which may suggest that those choosing to take a pain course may have had greater interest and/or background in the topic. However, baseline PAPS-PT scores on both subscales were not different between the pain elective and other elective at baseline (less than 1.5 points differences on each subscale).

While we advocate for comprehensive pain education curriculum that is threaded throughout pre-licensure programs, achieving these guidelines within existing programs may be challenging, requiring careful curricular mapping and potential restructuring of content [6]. Furthermore, successful integration requires faculty that have adequate knowledge and skills to teach the pain curriculum (neuromuscular, cardiopulmonary, neurology, pediatrics, and other special populations), which may require additional resources and to address this educational need. Therefore a comprehensive stand-alone course, in addition to integrated pain throughout a curriculum, may be a practical option for some existing programs.

Given the emergence of pain courses targeted at health care professionals [14, 32], we asked if previous pain education, i.e. exposure to pain education beyond pre-licensure curriculum, had an effect on students’ pain knowledge. Remarkably, 20% of the 3rd year students self-reported on other pain course attendance. Previous pain education did not significantly change mean scores on students’ knowledge or their attitudes and beliefs on pain when compared to those that had not taken supplemental courses. Given our small sample size (*n* = 11) and our inability to plan for a priori analysis on the students that had participated in these brief educational experiences, we cannot be confident that this will hold true in a larger sample. Nonetheless, in our sample there were no apparent effects on students’ knowledge or their attitudes and beliefs on pain when compared to those that had not taken supplemental courses. Reasons for this are unclear but may be related to a number of factors including attrition of information and/or related to the type, content of program, length of program etc. (3 h-15 h) [14, 32]. Although we did not inquire about specific educational participation, we were nevertheless surprised to discover that self-reported educational experiences did not impact NPQ or PABS-PT scores in our cohort. Future studies are underway to define the specific courses attended as “previous pain education” and determine the short and long impact on students’ pain knowledge and beliefs.

#### Limitations

A limitation of this study is that it was performed in a single educational program with small sample size, and thus the results may not be generalizable to other pre-licensure physical therapy programs. Another limitation is that there was no long-term follow-up to assess retention of knowledge and impact on attitudes and beliefs. Further studies are needed to investigate if these improvements in pain knowledge and attitudes and beliefs towards working with people in chronic pain are sustained. Although the questionnaires identified changes in knowledge and attitudes and beliefs of students following a comprehensive IASP based course, the instruments did not address the full breadth and scope of a 30 h pain course, including understanding of pain assessment, treatment, and clinical conditions. Future studies are underway to assess the impact of a comprehensive pain course on these important components of pain education of health professionals. Additionally, although changes following pain education are evident there is a need to investigate if these findings translate into performance based outcomes of student clinical skills [33, 34]. As greater implementation of the IASP guidelines is incorporated into pre-licensure programs, it will be important to investigate competency based education outcomes.

## Conclusion

The data reported here demonstrates final semester DPT students showed greater pain knowledge and a difference in their attitudes and beliefs on pain with greater biopsychosocial and less biomedical orientation compared to first semester DPT students. Additionally, students taking a separate pain elective showed improved knowledge, and most importantly, further shifted their beliefs towards patients with pain to a biopsychosocial orientation. We suggest that a stand-alone course on pain covering core competencies as outlined by IASP Guidelines [9, 13], as an addition to pain management concepts threaded throughout the curriculum, may enhance students understanding of pain. Given the impact of pain on society and the integral role that physical therapists play in pain management, realizing pain is a primary reason patients seek care by a physical therapist through referral by another practitioner or through direct access, physical therapy education should be at the forefront in training students on pain. The development of a stand-alone comprehensive pain education course may enhance knowledge of pain management, and ensure that entry-level clinicians are adequately prepared to optimize and direct treatments for patients with pain.

## Supplementary information

**Additional file 1:** **Table S1.** Comparison of original NPQ scores and revised NPQ scores excluding the 7 questionable items reported by Catley et al. 2013. NPQ scores are reported as percent correct, mean (standard error).

## Data Availability

The datasets used and/or analyzed during the current study are available from the corresponding author on reasonable request.

## Abbreviations

DPT: Doctorof Physical Therapy
IASP: International Association for the Study of Pain
NPQ: Neurophysiology of Pain Questionnaire
PABS-PT: Pain Attitudes and Beliefs Scale for Physiotherapists
SD: Standard deviation
