# Supplementary material for: Evaluation of pain knowledge and attitudes and beliefs from a pre-licensure physical therapy curriculum and a stand-alone pain elective
Source: BMC Med Educ. 2019 Oct 16;19:375. doi: 10.1186/s12909-019-1820-7 (PMC6796383; doi:10.1186/s12909-019-1820-7)
Supplement: Supplementary file 1 — Additional file 1: Table S1. Comparison of original NPQ scores and revised NPQ scores excluding the 7 questionable items reported by Catley et al. 2013. NPQ scores are reported as percent correct, mean (standard error). [file 12909_2019_1820_MOESM1_ESM.docx]

**Additional file 1: Table S1.** Comparison of original NPQ scores and revised NPQ scores excluding the 7 questionable items reported by Catley et al. 2013. NPQ scores are reported as percent correct, mean (standard error).

|  | 1^st^ YEAR | 3^rd^ YEAR,  Pre-Other Elective (OE) | 3^rd^ YEAR,  Pre-Pain Elective (PE) |
| --- | --- | --- | --- |
| N | 72 | 26 | 30 |
| NPQ Score | 64.0% (1.2) | 73.5% (1.8) | 79.8% (1.5) |
| Revised NPQ Score | 63.1% (1.4)  p= 0.82 | 72.4% (1.1)  p=0.29 | 83.0% (1.4)  p=0.68 |
